# Mitigation of Iron Irradiation-Induced Genotoxicity and Genomic Instability by Postexposure Dietary Restriction in Mice

**DOI:** 10.1155/2021/2888393

**Published:** 2021-11-25

**Authors:** Bing Wang, Takanori Katsube, Kaoru Tanaka, Masahiro Murakami, Mitsuru Nenoi

**Affiliations:** ^1^Dietary Effects Research Group, Department of Radiation Effects Research, National Institute of Radiological Sciences, Quantum Life and Medical Science Directorate, National Institutes for Quantum and Radiological Science and Technology, Chiba, Japan; ^2^Human Resources Development Center, Quantum Life and Medical Science Directorate, National Institutes for Quantum and Radiological Science and Technology, Chiba, Japan

## Abstract

*Background and Purpose*. Postexposure onset of dietary restriction (DR) is expected to provide therapeutic nutritional approaches to reduce health risk from exposure to ionizing radiation (IR) due to such as manned space exploration, radiotherapy, or nuclear accidents as IR could alleviate radiocarcinogenesis in animal models. However, the underlying mechanisms remain largely unknown. This study is aimed at investigating the effect from postexposure onset of DR on genotoxicity and genomic instability (GI) induced by total body irradiation (TBI) in mice. *Materials and Methods*. Mice were exposed to 2.0 Gy of accelerated iron particles with an initial energy of 500 MeV/nucleon and a linear energy transfer (LET) value of about 200 keV/*μ*m. After TBI, mice were either allowed to free access to a standard laboratory chow or treated under DR (25% cut in diet). Using micronucleus frequency (MNF) in bone marrow erythrocytes, induction of acute genotoxicity and GI in the hematopoietic system was, respectively, determined 1 and 2 months after TBI. *Results and Conclusions*. TBI alone caused a significant increase in MNF while DR alone did not markedly influence the MNF. DR induced a significant decrease in MNF compared to the treatment by TBI alone. Results demonstrated that postexposure onset of DR could relieve the elevated MNF induced by TBI with high-LET iron particles. These findings indicated that reduction in acute genotoxicity and late GI may be at least a part of the mechanisms underlying decreased radiocarcinogenesis by DR.

## 1. Introduction

Ionizing radiation (IR) as a carcinogen could induce genotoxicity, genomic instability (GI), and cancer. GI is an increased tendency to alterations in the genome and an important initiating and central event in carcinogenesis [1]. GI could be provoked by a variety of endogenous and exogenous insults including IR, and GI could be modified by lifestyle factors such as diet [2–6]. Characterized by various endpoints such as chromosomal rearrangements and aberrations, micronucleus formation, and gene mutation, IR-induced genotoxicity and GI have a big impact on radiocarcinogenesis. IR-induced GI is the driving force responsible for radiocarcinogenesis [1, 7–10]. Radiocarcinogenesis is the most concerned long-term health consequences. As humans are unavoidably exposed to high linear energy transfer (LET) IR in some circumstances such as hadrontherapy and manned space activities, limiting cancer risk from exposure to high-LET IR is of great public concern [11, 12].

Many factors could modify IR-induced biological effects including carcinogenesis, such as intervention of dietary and lifestyle-related factors. Studies show clearly that certain cancers are primarily dependent on dietary habits [13, 14], and dietary and lifestyle-related factors could influence health in many species, playing key roles in modulating the risk of developing cancer. It is known that dietary restriction (DR), i.e., restriction of either calories or macronutrients and fasting, could increase mean lifespan by decelerating aging rate and inhibiting tumor formation in a variety of species [15–17]. DR could decrease both spontaneous and chemical carcinogen-induced tumors in rodents and nonhuman primates [18–20]. DR could act synergistically with other treatments [21] and decrease significantly the incidence of both spontaneous and induced neoplasms in experimental carcinogenesis [21–26]. For prevention of radiocarcinogenesis, the early pioneer studies show that food or caloric restriction could decrease dramatically low LET gamma- or X-ray-induced solid tumors and/or leukemia in mice and rats [27–29]. DR could not only protect acute IR-induced damage and promote early regeneration [30] but also suppress residual genotoxic damage [31] and development of cancer including its initiation, progression, and metastasis [22]. Preexposure onset of caloric restriction could extend latency of myeloid leukemia and prevent radiation-induced myeloid leukemia and life shortening in mice [32–34]. Furthermore, postexposure onset of DR during the tumor promotion/progression phase could still be a valuable strategy in extending lifespan, reducing frequencies of radiocarcinogenesis for myeloid leukemia [35] and late-occurring tumor [36] and suppressing the size and progression of intestinal tumors [37]. All these findings demonstrate that DR including postexposure onset of DR could generally prevent incidence of radiocarcinogenesis in experimental models. On the other hand, the mechanisms underlying postexposure onset DR-induced reduction of radiocarcinogenesis are still largely unknown. A few of studies in mouse models show that suppression of radiocarcinogenesis could be attributed to the reduced IR-induced mutations [38, 39]. In this work, the impact of postexposure onset of DR on high-LET IR-induced acute genotoxicity and late GI was investigated in a mouse model measured as micronucleus erythrocytes in the bone marrow. We demonstrated that postexposure onset of DR could efficiently reduce acute genotoxicity and late GI in the erythrocytes in the bone marrow without significant change in peripheral blood hemogram. Our findings suggested that DR could activate mechanisms consequently resulting in suppression of IR-induced genotoxicity and GI.

Micronucleus formation resulted from DNA damage and defects in mitosis. It could serve as an index of genotoxicity and chromosomal instability [40]. Micronucleus is a highly accepted biomarker for the detection and quantification of GI to predict cancer risk and identify high risk individuals, and the micronucleus test is one of the most widely used assays to evaluate GI in different tissues [6, 41–46]. In this work, the impact from postexposure onset of DR (25% cut in diet) on genotoxicity and GI induced by high-LET iron irradiation was investigated in a mouse model measured as changes in the micronucleus frequency (MNF) in bone marrow erythrocytes, respectively, 1 and 2 months after total body irradiation (TBI). Results demonstrated that postexposure onset of DR could relieve the elevated MNF induced by high-LET iron irradiation.

## 2. Materials and Methods

### 2.1. Animals

Seven-week-old C57BL/6J Jms strain female mice were purchased from SLC, Inc. (Japan). To avoid possible effects from the developmental condition of the animals, any mouse with a significantly different body weight, namely, more or less than the mean ± 2 standard deviation (SD) of all the animals upon arrival, was omitted from this study. The selected mice were randomly assigned to 2 experimental groups as either the nonirradiated group or the irradiated group. All animals were maintained in a conventional animal facility under a 12 h light-12 h dark photoperiod, controlled temperature (23 ± 2°C), and humidity (50 ± 10%); housed in autoclaved aluminum cages (one mouse per cage) with sterilized wood chips; and allowed access to a standard laboratory chow MB-1 (Funabashi Farm Co., Japan) and acidified water (pH = 3.0 ± 0.2) *ad libitum*. Ingredients of the diet MB-1 contained 24.2% crude protein, 4.4% crude fat, and 54.4% carbohydrate. The metabolizable energy was 354.0 kcal/100 g. The mice were acclimatized to the laboratory conditions for 1 week before use. The mice at postnatal 8 weeks old in the irradiated group were irradiated with iron particles. Then, the animals in each of the experimental groups were further divided into 2 subgroups, namely, the Control group (Control, without radiation exposure and DR), the DR group (DR, receiving the 25% cut in diet), the exposure group (2.0 Gy, receiving a total body exposure to iron particles at a dose of 2.0 Gy), and the exposure plus DR group (2.0 Gy + DR, receiving a total body exposure to iron particles at a dose of 2.0 Gy plus the 25% cut in diet). The animals under DR were given daily (around 9:30 am) 75% of the amount (weight in gram) of the chow consumed by the animals that were allowed to free access to the diet. The mean amount of chow consumed per mouse allowed free access to the diet was 2.92 g per day; each of the mice under the 25% cut in diet was given daily 2.19 g of the chow. Thus, the weekly metabolizable energy was 72.36 kcal and 54.27 kcal, respectively, for each of the mice without DR and under DR. All animals were allowed access to the acidified water *ad libitum*. Based on our previous studies and preliminary trials, in the present study, 20-24 mice were used in each experimental subgroup.

All experimental protocols (Experimental Animal Research Plan No. 09-1049-1, No. 09-1042 and No. 17-2006) involving mice mentioned above and descripted in the irradiation section were reviewed and approved by The Institutional Animal Care and Use Committee of the National Institute of Radiological Sciences, National Institutes for Quantum and Radiological Science and Technology. The experiments were performed in strict accordance with the Institutional Guidelines for the Care and Use of Laboratory Animals.

### 2.2. Irradiation

Iron particles were generated and accelerated by a synchrotron, the Heavy Ion Medical Accelerator in Chiba (HIMAC), Japan. The monoenergetic iron beams having 500 MeV/nucleon of initial energy were expanded by wobbler magnets to a 10 cm irradiation field with homogeneous irradiation dose. Animals were irradiated at the entrance (plateau) region of the iron beams. The dose-averaged LET value of iron particles calculated by the Monte-Carlo simulation was 200 ± 20 KeV/*μ*m. TBI with 2.0 Gy was performed at a dose rate about 1.0-2.0 Gy/min. For TBI, the mice were held in a special Lucite columnar container, which was with an outer diameter of 10 cm and 3 individual cells of the same size (each mouse in each cell). The mice were in an air-breathing condition (there were six holes 5 mm in diameter in the wall of each cell). The containers were set on the beam track, and the focused 10 cm diameter iron beam was delivered to the animals at room temperature without anesthesia.

### 2.3. Micronucleus Test

The micronucleus test has been extensively used in a variety of exploratory and mechanistic studies aiming to explore the mechanisms underlying genotoxicity. Due to its simplicity and readiness, this test could be applied to a variety of cell types. An increase in the micronucleus frequency in treated animals is an indication of induced chromosome damage. A bone marrow erythrocyte micronucleus test was carried out according to our previous study [31]. Induction of micronucleus erythrocytes in bone marrow by TBI was used as an index to evaluate radiation-induced acute genotoxicity and late GI, depending on early and late timing of measurement after exposure. Mice were sacrificed by CO_2_ asphyxiation 1 or 2 months after TBI. Bone marrow was collected from both femurs. Then, bone marrow smears were prepared and processed for the enumeration of micronucleated polychromatic erythrocytes (MNPCEs) and micronucleated normochromatic erythrocytes (MNNCEs). The slides were coded to avoid observer bias. The micronuclei were scored using a light microscope at a magnification of 1000x. At least 5000 PCEs and 5000 NCEs per mouse were counted, and the data for each experimental point were from at least 6 mice.

### 2.4. Physiological Endpoints

Physiological conditions were comparatively studied in mice that were allowed free access to the diet and being under DR. The assessments included evaluating changes in body mass and measurements of peripheral hemogram. For monitoring body weight gain, all the animals were weighed weekly from onset of DR at postnatal age 8 weeks (immediately after TBI) to the end of experiment at postnatal age 17 weeks (2 months after TBI). The body weight gain data for each experimental subgroup were from at least 10 mice. For the analysis of hemogram, animals were anesthetized by CO_2_ inhalation 1 or 2 months after TBI. The peripheral blood was collected from a femoral artery with a heparinized syringe in vacutainer blood collection tubes containing EDTA (Venoject II, Terumo Co., Japan), and the animals were killed by cervical dislocation. For the analysis of hemogram, blood samples were immediately subjected to a differential blood cell count and hemoglobin concentration measurement using a blood cell differential automatic analyzer (SYSMEX K-4500, Sysmex Corporation, Japan). The data for each experimental subgroup were from at least 6 mice.

### 2.5. Statistical Analysis

Statistical evaluation of the data was done with the chi-squared test for the micronucleus test and Student's *t*-test the other endpoints. The statistical significance was assigned to *P* < 0.05.

## 3. Results

### 3.1. Body Weight Gain

**Figure 1:**
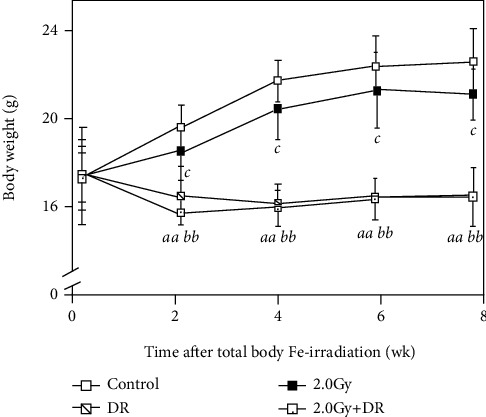
Effects of TBI and DR on body weight gain in mice. Body weight in grams (g) is presented as the mean ± SD. The solid line with open, solid, striped, and dotted boxes, respectively, stands for the Control group, the DR group, the 2.0 Gy group, and the 2.0 Gy + DR group. Letters *aa* and *bb* indicate statistically significant difference at *P* < 0.01 between the Control group and the 2.0 Gy group and between the DR group and the 2.0 Gy + DR group, respectively. Letter *c* stands for statistically significant difference at *P* < 0.05 between the Control group and the DR group.

As an important index, changes in body mass were assessed to evaluate physiological effects from TBI and DR (Figure 1). Although a tendency for body weight gain was observed in animals in both the Control group and the 2.0 Gy group, in the 2.0 Gy group, the body weight gain decreased markedly than that in the Control group. On the other hand, significant reduction of body weight gain in the DR group and the 2.0 Gy + DR group was observed regardless of TBI. Body mass measurements of animals under DR pointed to a general significantly lower body weight gain after onset of DR until the end of the experiment compared to that of animals without DR. In addition, animals under DR have no significant difference in physiological appearance compared to their counterparts and no mortality occurred throughout the whole monitoring period. Results indicate that DR has a big impact on body weight gain in mice during the whole period of diet regimen in our experimental setup.

### 3.2. Peripheral Blood Hemogram

**Figure 2:**
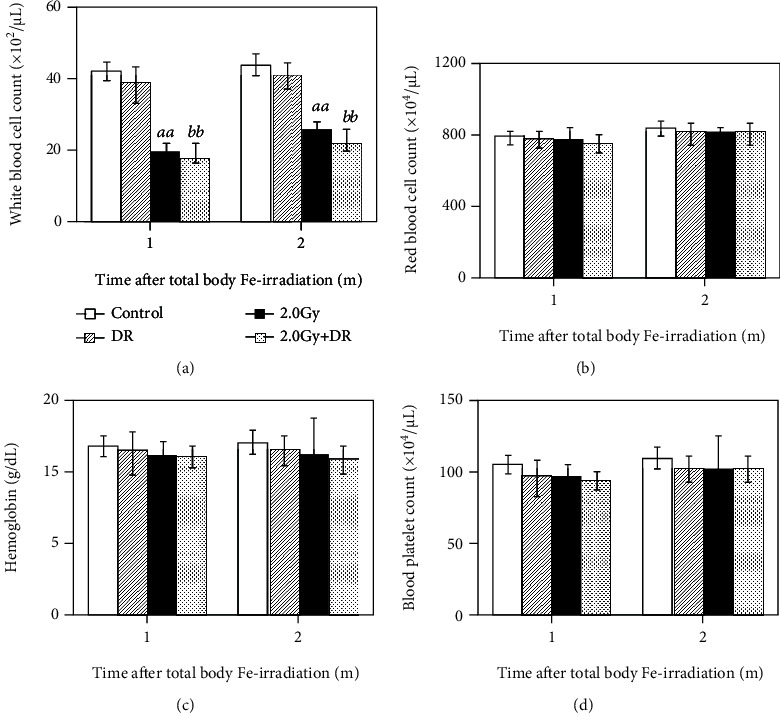
Effects of TBI and DR on peripheral blood hemogram in mice. (a) White blood cell count, (b) red blood cell count, (c) hemoglobin concentration, and (d) blood platelet count were measured 1 and 2 months after TBI. Cell count or hemoglobin concentration is presented as the mean ± SD. The open, solid, striped, and dotted bar stands for the Control group, the DR group, the 2.0 Gy Group, and the 2.0 Gy + DR group, respectively. Letters *aa* and *bb*, respectively, indicate statistically significant differences at *P* < 0.01 between the Control group and the 2.0 Gy group and between the DR group and the 2.0 Gy + DR group.

Alterations in the hematopoietic system measured as changes in peripheral blood hemogram were also studied to evaluate physiological effects (Figure 2). In general, DR alone did not induce any marked effects on all the parameters (Figure 2) while TBI alone significantly reduced the white blood cell count 1 and 2 months after exposure regardless of DR (Figure 2(a)). Though some alterations were detectable, no statistical significance was found in red blood cell count, hemoglobin concentration, and blood platelet count in animals from the groups treated with TBI, DR, or both, when compared to the Control group (Figures 2(b)-2(d)). These data clearly indicate that there is no significant alteration in peripheral blood hemogram in animals under DR compared to their counterparts without DR. On the other hand, TBI alone could induce significantly detrimental effects on the hematopoietic system measured as the persistent markedly lower white blood cell count in peripheral blood.

#### 3.2.1. Micronucleus Frequency

**Figure 3:**
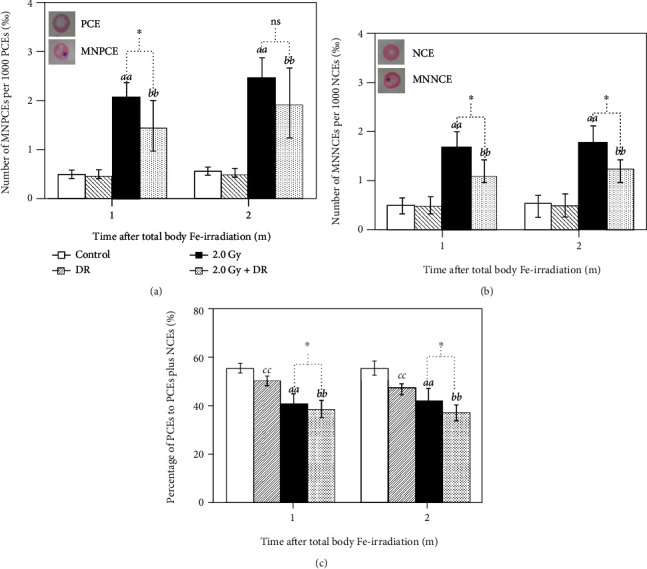
Effects of TBI and DR on induction of MNF and proliferation of bone marrow erythrocytes in the femur in mice. (a) The permillages (‰) of MNPCEs per 1000 PCEs and (b) MNNCEs per 1000 NCEs were used to measure MNF, and (c) the percentage (%) of PCEs to the sum of PCEs and NCEs as the indicator for proliferation. Letters *aa*, *bb*, and *cc*, respectively, indicate statistically significant difference at *P* < 0.01 between the Control group and the 2.0 Gy group, between the DR group and the 2.0 Gy + DR group, and between the Control group and the DR group. One asterisk (∗) stands for statistically significant difference at *P* < 0.05 between the 2.0 Gy group and the 2.0 Gy + DR group.

Induction of micronuclei measured as MNF in PCEs (Figure 3(a)) and NCEs (Figure 3(b)) was used to evaluate acute genotoxicity at 1 month after TBI and late GI at 2 months after TBI. In general, TBI alone could significantly increase the MNF in both PCEs and NCEs, namely, for MNF in PCEs in the 2.0 Gy group; it was 2.10 ± 0.33‰ and 2.50 ± 0.43‰, respectively, at 1 and 2 months after TBI while in the Control group it was 0.51 ± 0.08‰ and 0.57 ± 0.08‰; and for MNF in NCEs in the 2.0 Gy group, it was 1.70 ± 0.32‰ and 1.80 ± 0.33‰, respectively, at 1 and 2 months after TBI while in the Control group it was 0.48 ± 0.17‰ and 0.52 ± 0.18‰. On the other hand, although DR alone did not markedly change the MNF in the nonirradiated animals in the Control group, namely, for MNF in PCEs in the DR group, it was 0.50 ± 0.09‰ and 0.53 ± 0.09‰, respectively, at 1 and 2 months; and for MNF in NCEs in the DR group, it was 0.49 ± 0.19‰ and 0.50 ± 0.19‰. DR had a significantly inhibitory effect on induction of micronuclei by TBI, showing significantly decreased MNF in both PCEs and NCEs in the animals of the 2.0 Gy + DR group (for MNF in PCEs, it was 1.50 ± 0.51‰ and 1.97 ± 0.72‰, respectively, at 1 and 2 months; and for MNF in NCEs, it was 1.10 ± 0.34‰ and 1.25 ± 0.23‰) when compared to that in the 2.0 Gy group. Results clearly demonstrate that postexposure DR could relieve genotoxicity and GI caused by TBI with high-LET iron particles.

#### 3.2.2. Bone Marrow Proliferation

The percentage of PCEs to the sum of PCEs and NCEs, as an indicator for evaluating bone marrow cell proliferation condition, was assessed 1 and 2 months after TBI (Figure 3(c)). Proliferation in the animals after TBI (the 2.0 Gy group and the 2.0 Gy + DR group) was significantly inhibited manifesting as much lower percentages compared to that in the nonirradiated animals (the Control group and the DR group). On the other hand, all animals under DR regimen showed markedly decreased percentage when compared to their nonirradiated counterparts. Moreover, the percentages in the 2.0 Gy + DR group were significantly lower than that in the 2.0 Gy group 1 and 2 months after TBI. These findings indicate that either TBI or DR could decrease the proliferation of bone marrow cells, and concurrent exposure to both TBI and DR could further increase the inhibitory effect.

## 4. Discussion

Radiocarcinogenesis is one of the key concerns for medical, occupational, environmental, or accidental exposures to IR [47]. For example, exposure to high-LET IR during manned deep-space activities would increase unavoidably radiation health risk [12], and modern radiotherapy (RT) including high-precision hadrontherapy could control and cure efficiently cancers with high-LET IR while it still inevitably leads to increased mutation and secondary malignancy risk [11, 48]. Therefore, new methods for mitigating this adverse effect to limit cancer risk are urgently needed [11, 12]. GI is the earliest step and critical early event [1, 49–52], being central to carcinogenesis associating with cancer initiation and augmenting cancer progression [53–58]. It is the driving force responsible for radiocarcinogenesis [7–9]. In this work, we demonstrated that postexposure onset of DR could efficiently reduce acute genotoxicity and late GI measured as significantly decreased MNFs in PCEs and NCEs. Our findings suggested that DR could activate mechanisms consequently resulting in suppression of radiation-induced genotoxicity and GI.

Since the early work by Moreschi and McCay et al. [59, 60], the rapidly growing body of evidence on the effects from dietary intervention has shown that diet intervention has a great impact on health, pointing to a multitude of benefits affecting numerous physiological systems [61]. Diet intervention also elicits a variety of immediate and long-term physiological effects, in particular, beneficial effects on numerous diseases including cancer in experimental models [22]. Effects of DR depend on many factors including the restriction extent, dietary composition, and restriction onset timing. Mild DR (25% cut in diet) was considered adaptive and innocuous as DR at this level it was associated with increased longevity and decreased disease incidence in rodent models [62]. In a comprehensive study on the effects from various levels of food reduction on a wide range of toxicological parameters in rats, it also demonstrated that mild DR daily was dietary-optimized as a nutritionally appropriate and well-controlled animal model in conducting toxicity studies [63]. Based on a series of trials on the level of food reduction from 25% (mild DR) and 50% (moderate DR) to 75% (severe DR) of diet cut in the experimental conditions in our facilities, we also confirmed that mild DR was the dietary-optimized condition, and mild DR (25% cut in diet) was finally applied to the present study. Interestingly, in a series of investigations in rats and mice receiving DR, in the animals feed in amounts that limited the mean body weight to approximately 85% of the controls fed *ad libitum*, it was found that DR increased survival rates and decreased the incidences of chemical carcinogen-induced neoplasms and nonneoplastic lesions at a variety of sites [64]. In the present study, the extent of DR-induced body weight decrease was comparable to this work [64]. On the other hand, DR is effective on tumor initiation and more effective on tumor promotion phase [15, 65, 66]. In an analysis using a multistage carcinogenesis model [67] to the data obtained in a mouse lifespan study following postexposure onset of DR [36], it was shown that DR could offset both spontaneous and IR-induced carcinogenesis, and there is little or no interaction between the detrimental effects of IR and the beneficial effects of DR. DR could delay the onset of the tumors by overarchingly altering all the steps (i.e., decrease in the mutation rate) or a particular step necessary for carcinogenesis [38]. In the present work, we confirmed that postexposure onset of DR could also efficiently reduce IR-induced GI measured as decreased MNF.

The mechanisms underlying suppression by DR of radiocarcinogenesis including GI investigated in the present work are still largely unknown. Increased resistance to oxidative stress and enhanced DNA repair are possible mechanisms. It is known that exposure to high-LET iron significantly increased the oxidative stress (i.e., reactive oxygen species (ROS)) in the irradiated animals [68, 69]. Dietary intervention shows potential health benefits in humans and animals [70] as one of the means to minimize health risk from exposure to IR via increasing endogenous antioxidative protection [71, 72]. Studies showed that DR could lead to the reduction of oxidative damage to macromolecules [73], modulation of oxidative DNA damage, and enhancement of DNA repair via affection of adrenal metabolism, insulin metabolism, and various aspects of gene expression [16, 74]. DR could also decrease IR-induced mutation frequencies via suppression of oxidative stress in a radiocarcinogenesis model in mice [39]. Through a process known as mitohormesis, a retrograde response, DR could increase formation of ROS within the mitochondria. This could cause an adaptive response that culminates in subsequently increased stress resistance and ultimately lead to a long-term reduction of oxidative stress [75]. DR could further activate expression of endogenous antioxidant genes to produce ROS-eliminating enzymes and increase activities of antioxidant enzymes including superoxide dismutase, catalase, glutathione peroxidase, and paraoxonase [76, 77]. DR could also decrease oxidative protein modification and sensitivity of membranes to lipid peroxidation in association with a reprogramming of the respiratory chain complexes and apoptosis-inducing factor content [78] in experimental animal models. In addition, reduction (25% cut) in caloric intake could rapidly reduce and then sustain oxidative stress in humans [79].

In addition to DR-induced resistance to oxidative stress, postexposure DR-induced decrease in IR-induced genotoxicity and GI may be through shifting metabolism to less cell differentiation and proliferation and enhanced elimination of abnormal cells [80, 81]. Increased cell proliferation is associated with GI [82]. DR could decrease circulating levels of growth factors, anabolic hormones, inflammatory cytokines, and oxidative stress markers associated with various malignancies, exerting multiple suppressive effects on both target cells and microenvironments during carcinogenesis [83]. It is well known that dietary intervention was a regulator of stem cell behavior [84]. DR was able to restrict increase in hematopoietic stem and progenitor cells and formation of myeloid colony in mouse bone marrow [85]. Even a diet limited only in protein (4% cut by weight) for 3 weeks could markedly reduce hemopoietic stem cells in mice [86]. In the present work, our results clearly showed that DR could result in inhibition of erythrocytogenesis measured as decrease in the percentage of PCEs to the sum of PCEs and NCEs in the bone marrow in mice (Figure 3(c)). DR could also inhibit cell proliferation in spleen and thymus in mice [87]. Studies show that DR could induce memory T cell accumulation in bone marrow associating with enhanced protection against infections and tumors [88]. It was reported that calorie restriction (33% cut) could reduce cell proliferation in several tissues and cell populations including T cells within 2 weeks in mice that were of the same strain, sex, and age as used in the present work. This effect was potent and rapid and mediated anticarcinogenic effects [89]. It was also shown that moderate caloric restriction could contribute to slow down in aging and to prevent chronic diseases [90]. In murine models, studies showed that DR-induced delayed aging and retarded tumor development were attributed to induction of apoptosis to selectively eliminate preneoplastic and superfluous cells that negatively affected normal function and promote cell transformation [91]. Through metabolic energy modulation, DR could enhance autophagy [92].

There is a complex interplay among the diet, gastrointestinal microbiota, and health. One of the possible mechanisms underlying postexposure DR-induced mitigative effect on IR-induced genotoxicity and GI might be attributed to the DR-induced alterations in the mouse gastrointestinal microbiota. Diet is a key determinant of the microbiota diversity, composition, stability, activity, and function of gut. Gastrointestinal microbiota has a symbiotic relationship with the host and is involved in maintaining gastrointestinal homeostasis through its impact on nutrient metabolism, energy balance, gut barrier, inflammation, microenvironment, and immune and stress response [93–97]. The gastrointestinal microbiome plays a critical role in competitive pathogen exclusion and immune development. By influencing the immune system of the host, studies show that dietary intervention could change the composition or functions of the microbiota to confer health benefits including cancer prevention and treatment [93, 98–101]. Radiation exposure could disrupt the gut-brain axis [102]. On the other hand, diet contents and quantity could play a pivotal role in modifying susceptibility to IR exposure including both acute and late effects of radiation in animal models. As a therapeutic modality in various clinical contexts, gastrointestinal microbiota manipulation through dietary intervention is expected to maximize the response to treatment and minimize adverse effects by reducing radiation injury and improving the health in the treatment of accidental radiation exposure and restoration of human health from cancer RT [103–106].

Dietary and lifestyle factors are considered causes of cancer and targets for cancer prevention as well. As lifestyle and environmental factors could affect cancer initiation, promotion, and progression, radiocarcinogenesis could be preventable by intervention of lifestyle and environmental factors [83]. A healthy lifestyle (including but not limited to keeping a healthy diet and maintaining a healthy weight) is a simply efficient strategy for cancer prevention and reduction in cancer morbidity and mortality, and thus, dietary intervention should be given priority for cancer prevention [107–113], increasing resistance to chemotherapy and RT in normal cells and reducing certain side effects of cytotoxic therapy [114] and limiting tumor growth [115, 116]. We propose that a combination of DR and other cancer treatments (i.e., chemotherapy and RT) represents a potential strategy to increase the treatment efficacy and prevent IR risk in humans. In addition to the possible mechanisms discussed, there are still many questions to consider in further studies; for examples, how long does DR need to be imposed to be effective and what role does dietary composition play [117]? Further investigation is warranted to characterize the mitigative effects on IR-induced genotoxicity and GI by postexposure onset of DR and to explore the exact underlying mechanisms. Although challenges and remaining unknowns persist and need in-depth exploration, it is clear that multiple approaches can be applied simultaneously to obtain integrated results.

IR is widely used in a variety of fields such as applications in industry, agriculture, medicine, life science, and research. Simultaneously, IR also presents a potential health risk by causing health consequences including cancer. With the advances in hadrontherapy development and space exploration, humans now encounter increasing exposure to high-LET IR. Radiation protection is defined as the protection of people from harmful effects due to exposure to IR and the means for achieving this goal. Avoidance or reduction of exposure dose using the simple protective measures of time, distance and shielding is fundamental. Being different from the existing exposure mainly due to naturally occurring radioactive materials that exist in the environment and the planned exposure such as in occupational and therapeutical situations where radiation protection could be planned in advance, emergency exposure usually occurs in unexpected situations such as emergency nuclear events and thus requires urgent protective actions. Nowadays, research on radiation protection marks an exciting new era with novel endeavor and achievement of applications. The time comes to conceive a timely concept for radiation protection to further include multimodality treatments with multidisciplinary management to increase radioresistance and/or decrease radiosusceptibility, to prevent, mitigate, and treat radiation damage and reduce radiation health risk in both the individual and the group. A new concept “proactive radiation protection” has been proposed, which is conceived as vigorously implementing what we already know from transdisciplinary research in preventing radiation damage and reducing radiation health risk to proactively achieve radiation protection potential through medical intervention [118]. For example, cancer treatment will not be successfully accomplished devoid of multimodality treatments including proactive radiation protection. Cancer RT advanced in both methodology and biology is the chief nonsurgical method to control cancer while one of its major drawbacks is the development of secondary malignancies. To surmount this side effect, it needs to brace every nerve for a supreme effort. Multimodality treatments include not only therapeutic administration of radiation and pharmaceutical agents such as tissue-specific radiosensitizers and radioprotectors [11, 119–121] but also prospective application of gene therapy (i.e., to modulate target gene splicing or aberrant splicing isoforms) [122], induction of hormesis and adaptive response [123, 124], lifestyle intervention (including but not limited to such as dietary and nutritional interventions and administration of DR mimetic drugs) [114, 125, 126], psychiatric management [127], and other components such as public health issues. Among the treatments, dietary intervention as one of the adjuvant therapies is with a high acceptability and feasibility [128]. A big impact from DR on the health consequences of the cancer patient receiving RT is expected [129]. Postexposure onset of DR is within the scope of proactive radiation protection. Elucidation of the underlying mechanisms would be expected to connect to a pharmaceutical treatment, which would be easier to be emotionally acceptable and clinically practical.

## 5. Conclusion

Exposure of mice to TBI from high-LET iron particles caused a significant increase in MNF in bone marrow erythrocytes. Postexposure DR alone did not markedly influence the MNF in the nonirradiated mice but induced a significant decrease in MNF in the exposed animals when compared to that exposed to TBI alone. Postexposure onset of DR could relieve the elevated MNF induced by TBI, which is expected as one of the proactive strategies to prevent radiocarcinogenesis and achieve maximum benefit for the cancer patients receiving RT and the exposed victims in nuclear accidents. Reduction in acute genotoxicity and late GI may be at least a part of the mechanisms underlying decreased radiocarcinogenesis by DR.

## Data Availability

Data supporting the findings of the present study are available within the article.

## Abbreviations

DR: Dietary restriction
GI: Genomic instability
HIMAC: The Heavy Ion Medical Accelerator in Chiba
IR: Ionizing radiation
LET: Linear energy transfer
MNF: Micronucleus frequency
MNNCE: Micronucleated normochromatic erythrocyte
MNPCE: Micronucleated polychromatic erythrocyte
RT: Radiotherapy
SD: Standard deviation
TBI: Total body irradiation.
